# A saliva-derived metabolic signature with multi-omics support for oral squamous cell carcinoma diagnosis

**DOI:** 10.3389/fcell.2026.1901210

**Published:** 2026-09-25

**Authors:** Hongyu Zheng, Shuijuan Hu, Pingping Tian, Jing Xu, Yaqing Ma, Yuelian Liu, Kaitong Wei, Qiang Sun

**Affiliations:** 1 National Engineering Laboratory for Internet Medical Systems and Applications, The First Affiliated Hospital of Zhengzhou University, Zhengzhou, Henan, China; 2 Department of Oral and Maxillofacial Surgery, The First Affiliated Hospital of Zhengzhou University, Zhengzhou, Henan, China; 3 Department of Oral Cell Biology, Academic Center for Dentistry Amsterdam (ACTA), Vrije Universiteit Amsterdam and University of Amsterdam, Amsterdam, Netherlands; 4 Department of Oral and Maxillofacial Head and Neck Oncology, Shanghai Ninth People’s Hospital, Shanghai Jiao Tong University School of Medicine, Shanghai, China

**Keywords:** biomarker, oral squamous cell carcinoma, ROC, salivary metabolomics, single-cell RNA sequencing, transcriptomics

## Abstract

**Background:**

Oral squamous cell carcinoma (OSCC) lacks practical and non-invasive biomarkers for case detection. Saliva is an attractive source for biomarker discovery; however, many reported candidates lack support from independent biological data layers.

**Methods:**

We performed untargeted salivary metabolomics in 40 patients with OSCC and 40 healthy controls. Differential metabolites were identified using multivariate and univariate analyses. Candidate diagnostic metabolites were prioritized by support vector machine-recursive feature elimination, random forest, and receiver operating characteristic (ROC) analyses. The biological context of the salivary findings was further examined using two GEO transcriptomic cohorts, the TCGA-HNSC cohort, a single-cell RNA sequencing dataset, and public protein-level resources including the Human Protein Atlas and CPTAC.

**Results:**

OSCC and control samples showed distinct salivary metabolic profiles, and 19 annotated endogenous differential metabolites were retained for downstream analysis. These alterations were mainly enriched in amino acid- and lipid-related pathways, including arginine and proline metabolism. Within the discovery cohort, internal cross-validation and feature ranking converged on a seven-metabolite panel; however, because feature selection and evaluation were performed on the same 80 samples without nested cross-validation, performance estimates are subject to optimism bias and are not reported here as validated diagnostic metrics. In GSE6631 and GSE37991 showed concordant downregulation of linoleic acid metabolism, arachidonic acid metabolism, bile acid-related programs, fatty acid metabolism, and peroxisome pathways in tumor tissue. Single-cell analysis further suggested reduced activity of curated metabolic modules in malignant cells and tumor-associated tissue states, though these comparisons did not account for patient-level dependence. At the protein level, ALDH1A1 and VDR were decreased, whereas SLC7A11 was increased in tumor tissue.

**Conclusion:**

OSCC was associated with a saliva-derived metabolic signature supported by pathway-level concordance across multiple public data layers. The overall pattern suggests lipid remodeling, reduced bile acid-related signaling, altered amino acid metabolism, and enhanced redox defense. These findings support further targeted quantification and external validation of saliva-based biomarker candidates for OSCC, while the current panel should be regarded as a preliminary discovery-set candidate signature.

## 1 Introduction

Oral squamous cell carcinoma (OSCC) is the predominant malignancy of the oral cavity and accounts for more than 90% of oral cancers (Muralidharan et al., 2025). According to GLOBOCAN estimates, oral cancer continues to impose a substantial global health burden, with high incidence and mortality worldwide (Bray et al., 2024; Sung et al., 2021). Despite progress in surgery, radiotherapy, and systemic treatment, the overall 5-year survival rate of OSCC remains unsatisfactory at approximately 50%–60% (Johnson et al., 2020; Chow, 2020). This limited improvement is largely related to delayed diagnosis, as a considerable proportion of patients still present with locoregionally advanced disease. By contrast, patients diagnosed at an early stage have substantially better outcomes (Johnson et al., 2020). Earlier and more accessible detection strategies therefore remain a clinical priority.

Histopathological evaluation of biopsy specimens is still the diagnostic gold standard for OSCC, but its use is constrained by invasiveness, sampling bias, procedural discomfort, and limited feasibility for serial assessment. Conventional oral examination is widely used for screening, yet its sensitivity is variable and influenced by clinical experience and practice setting (Johnson et al., 2020). These limitations have driven growing interest in non-invasive biomarkers that are objective, repeatable, and clinically practical. Among candidate biofluids, saliva is especially appealing because sample collection is simple and non-invasive, the fluid is in direct contact with oral lesions, and it contains diverse molecules originating from both local tissues and systemic circulation (Li et al., 2004; Wong, 2012).

Saliva-omics integrates the analysis of salivary DNA, RNA, proteins, and metabolites for disease detection and monitoring (Wong, 2012). Among these components, metabolomics is particularly relevant because metabolites directly reflect downstream biological activity and may capture disease-associated functional changes more closely than upstream molecular layers (Pavlova and Thompson, 2016). Sugimoto et al. first provided proof of concept that salivary metabolic profiling could discriminate patients with oral and other cancers from healthy controls (Sugimoto et al., 2010). Subsequent work further supported the diagnostic potential of this approach in OSCC, including a study by Song et al. combining salivary metabolite profiling with machine learning for case-control discrimination (Song et al., 2020). Consistent with these observations, a systematic review reported strong diagnostic performance of LC-MS-based salivary metabolomics, and a recent meta-analysis yielded pooled sensitivity and specificity of 85% and 82%, respectively (Nijakowski et al., 2022; Nazar et al., 2024). Across studies, recurrent alterations have involved amino acid metabolism, glycolysis-related metabolites, lipids, polyamines, and choline-containing compounds, supporting the view that OSCC-associated metabolic reprogramming is reflected in saliva (Sugimoto et al., 2010; Broadfield et al., 2021).

The biological basis of these salivary metabolic changes is likely linked to metabolic reprogramming within the tumor itself. TCGA studies of head and neck squamous cell carcinoma have defined major genomic alterations and molecular subtypes associated with disease behavior and prognosis (Cancer Genome Atlas, 2015). In parallel, GEO based transcriptomic analyses have identified consistent dysregulation of pathways related to glycolysis, glutamine metabolism, and lipid metabolism in OSCC (Wang et al., 2022; Peng et al., 2023). Single-cell RNA sequencing has revealed invasive and heterogeneous malignant cell states in head and neck cancer (Puram et al., 2017), while more recent work has emphasized marked metabolic heterogeneity across cellular and spatial contexts (Cheng et al., 2024). Together, these datasets provide a useful framework for interpreting salivary metabolic alterations in relation to tumor biology.

However, the clinical relevance of salivary metabolites in OSCC has not yet been examined within a unified multi-omics framework. It remains unclear whether the metabolic pathways implicated by salivary metabolite changes show pathway-level concordance with tumor transcriptomic data and how these signals map onto cellular and protein-level contexts. Previous studies have expanded multi-omics research in OSCC (Gupta et al., 2024; He et al., 2023), and integration of metabolomics with transcriptomics has also been explored in oral cancer models (Nair et al., 2026). Nevertheless, a framework linking salivary metabolomics with GEO-based transcriptomic validation, TCGA survival analysis, and single-cell mapping is still lacking. In the present study, we integrated untargeted salivary metabolomics, transcriptomic analysis, survival-associated gene analysis, and single-cell pathway mapping to examine whether salivary metabolic alterations were directionally consistent with tumor-associated metabolic reprogramming. Through this approach, we sought to identify saliva-derived candidate biomarkers for OSCC case-control discrimination and to place them in a broader biological context. The study is exploratory in nature and does not include an independent validation cohort.

## 2 Methods

### 2.1 Study participants

This study was conducted at the First Affiliated Hospital of Zhengzhou University between January 2024 and May 2024. Forty patients with oral squamous cell carcinoma (OSCC) and 40 healthy controls who underwent routine health examination at the same hospital were enrolled. All participants provided written informed consent. Complete clinical and pathological data were available for all subjects. The study was approved by the Ethics Committee of the First Affiliated Hospital of Zhengzhou University (Ethics Approval No. 2023-KY-1160).

The inclusion criteria for the OSCC group were as follows: (1) histopathological confirmation of OSCC; (2) no severe periodontitis, advanced dental caries, or other serious oral diseases within 3 months before enrollment; (3) no severe systemic disease; (4) no previous surgery; and (5) no history of antibiotic use, radiotherapy, or chemotherapy before enrollment. The exclusion criteria for the OSCC group were as follows: (1) oral infectious disease or oral bleeding history; (2) antibiotic use within 3 months before enrollment; and (3) other systemic diseases.

The inclusion criteria for healthy controls were as follows: (1) no antibiotic use within 3 months before enrollment; (2) no severe periodontitis, advanced dental caries, or other serious oral diseases; and (3) no personal or family history of autoimmune disease or other severe systemic disease. Subjects who did not sign informed consent were excluded.

### 2.2 Sample collection and pretreatment

Saliva samples were collected with reference to a previously reported protocol for patients with OSCC (Stephen and Speirs, 1976; Navazesh, 1993). Sample collection was performed between 8:00 and 9:00 a.m. All participants were asked to avoid smoking, alcohol consumption, and food intake for at least 1 h before sampling. Before collection, each participant rinsed the mouth three times with normal saline. Participants were then instructed to gently place the tip of the tongue against the palate or the lingual side of the mandibular incisors to promote saliva secretion. Unstimulated saliva was slowly collected into centrifuge tubes until 5 mL of bubble-free saliva was obtained. Saliva samples were centrifuged at 1000 *g* for 10 min at 4 °C. The supernatant was transferred into fresh EP tubes. After coding, all samples were stored immediately at −80 °C until further analysis.

For metabolomic pretreatment, 500 μL saliva supernatant was mixed with precooled methanol containing the internal standard for protein precipitation. After vertexing, the mixture was kept at −20 °C for 30 min. The samples were then centrifuged at 13,500×g for 15 min at 4 °C. The supernatant was collected and dried under nitrogen. The residue was reconstituted in acetonitrile: water (1:1), vortexed, and centrifuged again at 13,500×g for 10 min. The final supernatant was used for LC-MS analysis. Quality control (QC) samples were prepared by pooling equal volumes of supernatants from all samples. All pretreatment procedures were performed on ice or at low temperature to minimize metabolite degradation.

### 2.3 LC-MS analysis and data acquisition

Chromatographic separation was performed on a Dionex UltiMate 3000 liquid chromatography system (Thermo Scientific, San Jose, CA, United States). An ACQUITY UPLC BEH C18 column (2.1 mm × 100 mm, 1.7 μm; Waters, United States) was used. Mobile phase A was acetonitrile, and mobile phase B was water containing 0.1% formic acid. Gradient elution was applied: the injection volume was 5 μL, the column temperature was set at 40 °C, the flow rate was 0.35 mL/min.

Mass spectrometry was performed on a Q Exactive Orbitrap mass spectrometer (Thermo Fisher Scientific, San Jose, CA, United States). The scan range was 80–1200 m/z, the ion source temperature was 350 °C. The capillary temperature was 320 °C, collision energies were set at 20, 40, and 60 eV. Spray voltages were +3.5 kV in positive ion mode and −2.8 kV in negative ion mode. Data were acquired in full MS/dd-MS2 mode under both positive and negative ion conditions.

QC samples were prepared by mixing all saliva samples and were processed in the same way as the test samples. Experimental samples were analyzed in random order. One QC sample was injected after every 10 samples to monitor instrument stability. Mixed standard solutions were analyzed using the same procedure as biological samples. Raw mass spectrometry data were acquired with Xcalibur software (Thermo Fisher Scientific, United States). Metabolite information, including molecular weight, retention time, and peak area, was extracted with Compound Discoverer 2.1 software. Metabolites were annotated using the Human Metabolome Database (HMDB, http://www.hmdb.ca/).

### 2.4 Metabolomic data analysis and visualization

Univariate analysis was first used to screen differential metabolites. Multivariate statistical analyses, including principal component analysis (PCA) and orthogonal partial least squares-discriminant analysis (OPLS-DA), were then carried out using SIMCA software (version 14.0). Permutation testing was used to evaluate OPLS-DA model stability. Features with variable importance in projection (VIP) values greater than 1 were further prioritized, and the final set of annotated endogenous differential metabolites was used for downstream visualization and interpretation. Results were visualized using score plots, volcano plots, and heatmaps.

### 2.5 Construction of the diagnostic metabolite model

To evaluate the discriminatory potential of the differential metabolites, support vector machine-recursive feature elimination (SVM-RFE), random forest ranking, binary logistic regression, and receiver operating characteristic (ROC) analysis were used. Candidate metabolites were prioritized by overlap across the ranking approaches and individual ROC performance. Feature selection and model evaluation were performed on the same discovery cohort (n = 80) without nested cross-validation; therefore, all performance estimates should be interpreted as discovery-set internal evaluation only. No independent salivary validation cohort was available for this study. Performance metrics such as AUC, sensitivity, and specificity are subject to optimism bias when feature selection and evaluation share the same samples; accordingly, these metrics are not reported as validated diagnostic estimates. The final multi-metabolite panel is presented as a candidate discriminatory signature, and its clinical utility cannot be assessed without targeted quantification and external validation.

### 2.6 Pathway scoring and functional enrichment analysis

The GSVA package was used to perform single-sample gene set enrichment analysis (ssGSEA) and to calculate pathway activity scores. The reference gene sets included the Hallmark, Gene Ontology (GO), Kyoto Encyclopedia of Genes and Genomes (KEGG), and Reactome databases. Group comparisons of pathway scores were interpreted with attention to multiple-testing burden. Both raw P values and Benjamini–Hochberg false-discovery-rate-adjusted q values are reported in the supplementary tables; findings that survive FDR correction (q < 0.05) are distinguished from nominal trends throughout the Results. Functional enrichment analysis was performed with the clusterProfiler and, the results were visualized using ggplot2.

### 2.7 Survival analysis

Transcriptomic and survival data of patients with head and neck squamous cell carcinoma (HNSC) were downloaded from The Cancer Genome Atlas (TCGA). Patients were divided into high-score and low-score groups according to pathway scores. The optimal cut-off value was determined with the survminer package. Kaplan-Meier survival analysis was then performed based on this cut-off. A two-sided P < 0.05 was considered statistically significant. Because TCGA-HNSC includes multiple anatomic subsites beyond the oral cavity, this cohort was treated as a heterogeneous pan-HNSC reference rather than as an OSCC-specific validation dataset. Survival analyses were considered exploratory and are presented with cautious interpretation.

### 2.8 Single-cell transcriptomic analysis

Single-cell transcriptomic data were obtained from the GSE181919 dataset in the Gene Expression Omnibus (GEO) database. The UMI counts and barcode metadata files were downloaded, quality control, normalization, dimensionality reduction, and clustering were performed using the Seurat package. Cells with mitochondrial gene percentage (percent.mt) > 10% were removed. Doublets were predicted and filtered using the Singlet package. Marker genes for each cluster were identified using the FindMarkers function (logfc.threshold = 0.25, p_val_adj<0.05). Cell subtypes were annotated according to marker genes reported in the original study.

Cellular metabolic activity was evaluated using the SCPA package (Bibby et al., 2022). The metabolic pathway gene sets are listed in Supplementary Table 1. The AUCell function was used to calculate activity scores for five hypothesis-guided metabolic modules selected to summarize major biological themes suggested by the salivary metabolite findings. The corresponding gene lists are provided in Supplementary Table 2, enabling readers to independently evaluate module composition and to reproduce the analysis using alternative pathway definitions. Violin plots were used to display pathway score differences across cell populations or groups. Pathway comparisons were performed at the individual cell level. Patient-level dependence was not modeled through pseudobulk aggregation or mixed-effects approaches; therefore, the single-cell pathway analyses should be interpreted as exploratory and descriptive rather than as formal confirmatory tests.

### 2.9 Protein expression and immunohistochemical validation

To provide orthogonal protein-level context for selected pathway-related genes, protein data for ALDH1A1, SLC7A11, and VDR were retrieved from the Human Protein Atlas (HPA) and CPTAC resources. HPA immunohistochemical images were used as qualitative contextual evidence, whereas CPTAC-derived protein abundance data were used for cohort-level comparison between tumor and normal tissues.

### 2.10 Statistical analysis

All statistical analyses were performed in R software (version 4.4.3). Continuous variables were compared using the Wilcoxon rank-sum test, and categorical variables were compared using the chi-square test or Fisher’s exact test, as appropriate. Unless otherwise specified, all tests were two-sided. Where multiple comparisons were performed, Benjamini–Hochberg false-discovery-rate-adjusted q values are reported alongside raw P values in the supplementary tables; in the main text, results are described with their adjustment status explicitly noted. Correlation analyses used Spearman’s rank correlation.

## 3 Results

### 3.1 Overall study design and multi-omics workflow

We used a stepwise workflow to define saliva-derived metabolic features in OSCC and to assess their biological relevance across multiple data layers. We first performed untargeted salivary metabolomics in patients with OSCC and healthy controls, the baseline data of these subjects are shown in Table1. We then prioritized candidate discriminatory metabolites using machine-learning-based ranking and ROC analysis, all within the discovery cohort. Next, we examined related pathway changes in two GEO cohorts and in TCGA-HNSC as a heterogeneous reference dataset. Single-cell RNA sequencing data were used to assess module activity across tissue states and cell populations. Finally, representative pathway related proteins were evaluated with HPA immunohistochemistry and CPTAC proteomic data (Figure 1).

**TABLE 1:** Demographic and clinical baseline characteristics of OSCC patients and healthy controls.

Variable	OSCC patients (n = 40)	Healthy controls (n = 39)	Statistic	P value
Age (years)	58.5 (48.2, 68.2)	59.0 (46.0, 65.5)	U = 726.5	0.603
Sex	​	​	χ^2^ = 0.312	0.576
Male	22 (55.0%)	19 (48.7%)	​	​
Female	18 (45.0%)	20 (51.3%)	​	​
BMI (kg/m^2^)	23.94 ± 3.74	24.64 ± 2.60	t = 0.970	0.335
Smoking	​	​	χ^2^ = 0.735	0.391
Yes	16 (40.0%)	12 (30.8%)	​	​
No	24 (60.0%)	27 (69.2%)	​	​
Alcohol consumption	​	​	χ^2^ = 2.484	0.115
Yes	8 (20.0%)	14 (35.9%)	​	​
No	32 (80.0%)	25 (64.1%)	​	​
Betel nut chewing	​	​	Fisher	0.055
Yes	5 (12.5%)	0 (0.0%)	​	​
No	35 (87.5%)	39 (100.0%)	​	​
Clinical characteristics (patients only)				
Tumor site				
Tongue	17 (42.5%)	​	​	​
Gingiva	12 (30.0%)	​	​	​
Buccal mucosa	8 (20.0%)	​	​	​
Other	3 (7.5%)	​	​	​
T stage	​	​	​	​
T1	12 (30.0%)	​	​	​
T2	26 (65.0%)	​	​	​
T3	2 (5.0%)	​	​	​
N stage				
N0	17 (42.5%)	​	​	​
N1	4 (10.0%)	​	​	​
N2	10 (25.0%)	​	​	​
Nx	9 (22.5%)	​	​	​
M stage (M0)	40 (100.0%)	​	​	​
Lymph node metastasis (yes)	11 (27.5%)	​	​	​
Perineural invasion (yes)	10 (25.0%)	​	​	​

Data are presented as n (%) for categorical variables, median (interquartile range) for age, and mean ± SD, for BMI., Age was compared by the Mann–Whitney U test; BMI, by Welch’s t-test; sex, smoking, and alcohol consumption by the chi-square test; and betel nut chewing by Fisher’s exact test. Abbreviations: BMI, body mass index; OSCC, oral squamous cell carcinoma; SD, standard deviation.

**FIGURE 1:**
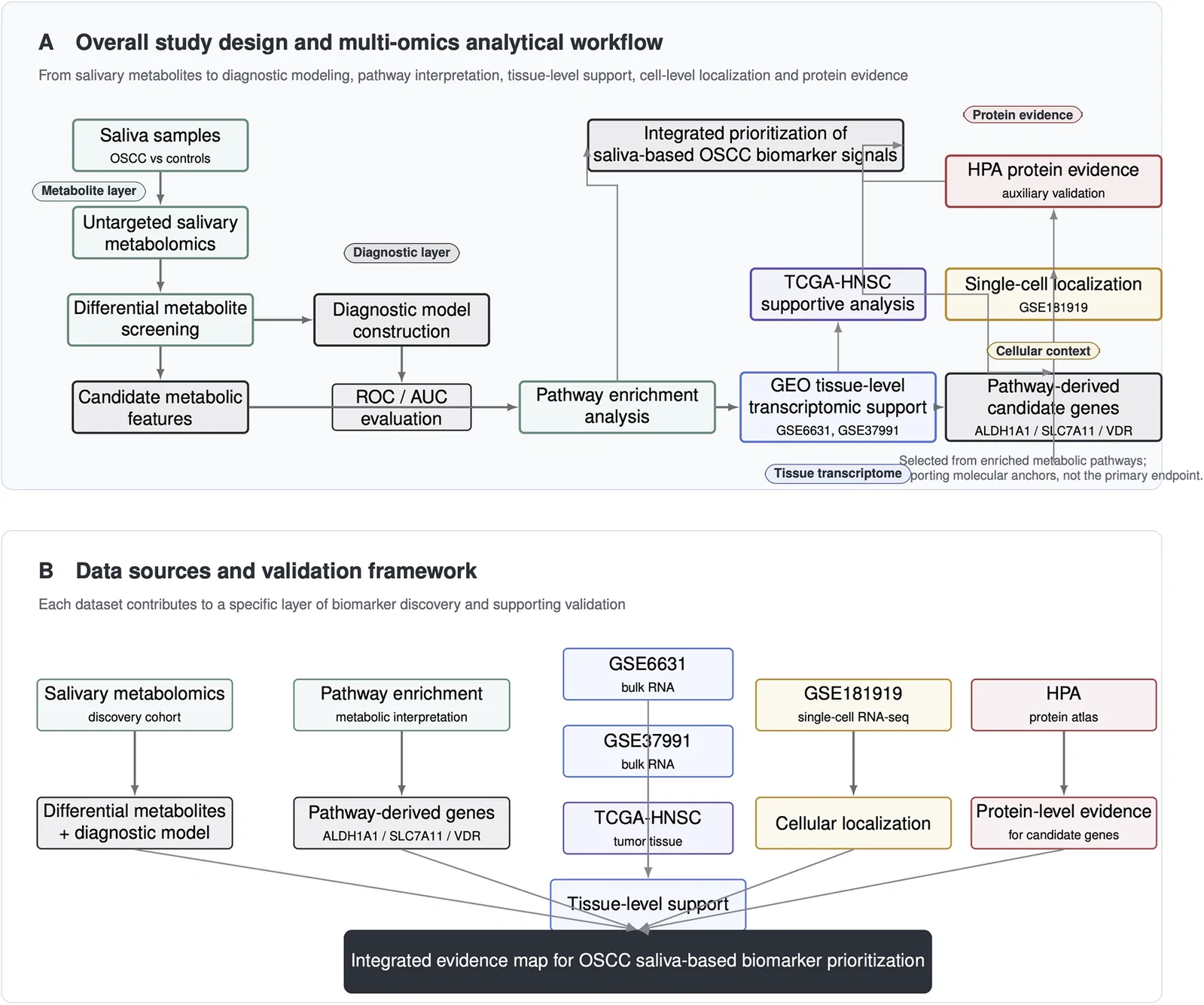
Overall study design and multi-omics workflow. Schematic overview of the study design. Untargeted salivary metabolomics was performed to identify differential metabolites between OSCC and healthy controls. Candidate diagnostic metabolites were selected by machine learning and ROC analysis. Pathway-related changes were then examined in two GEO transcriptomic cohorts and in the TCGA-HNSC dataset. Single-cell RNA sequencing was used to assess the activity of curated metabolic modules across tissue states and cell populations. HPA immunohistochemistry and CPTAC proteomic data were used for protein-level validation.

### 3.2 Salivary metabolomic profiling revealed a distinct metabolic pattern in OSCC

The salivary metabolic profiles of the OSCC and control groups showed clear separation at the global level (The identification results of all metabolites are provided in Supplementary Table 1). QC samples clustered tightly, supporting assay stability during data acquisition. PCA indicated a shift in the overall metabolic landscape, and OPLS-DA further increased group separation. Permutation analysis supported model stability at the profiling stage, although this should not be interpreted as independent validation of the diagnostic classifier (Figure 2).

**FIGURE 2:**
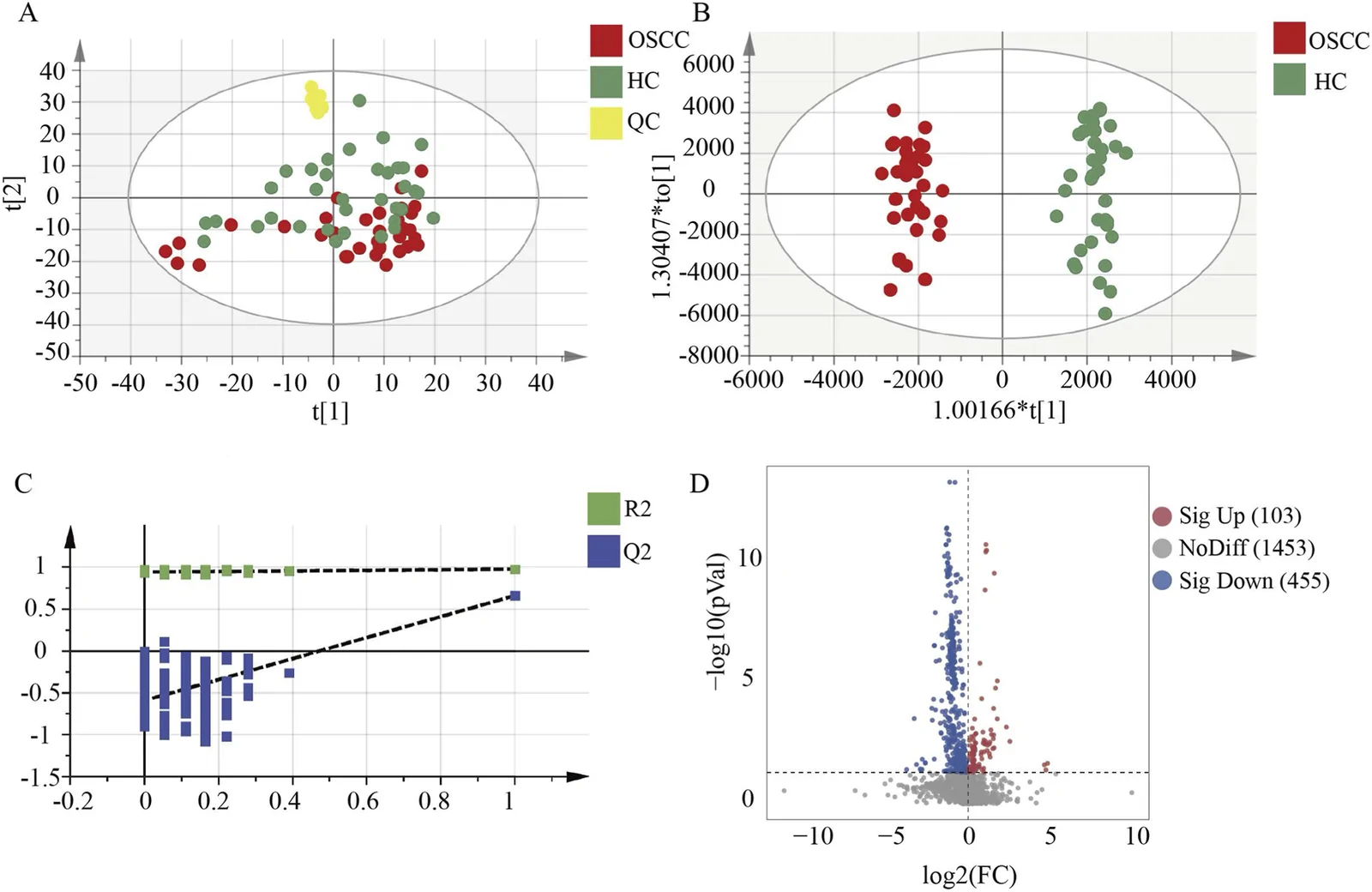
Salivary metabolomic profiling revealed clear separation between OSCC and healthy controls. **(A)** PCA score plot of the salivary metabolomic data, showing separation between OSCC and healthy controls and tight clustering of QC samples. **(B)** OPLS-DA score plot showing improved group discrimination. **(C)** Permutation analysis of the OPLS-DA model, supporting model robustness and arguing against major overfitting. **(D)** Volcano plot of differential metabolite features between OSCC and healthy controls.

The heatmap showed a coordinated pattern across the two groups rather than scattered single-metabolite changes (Figure 3A). After preprocessing, statistical screening, and endogenous-metabolite curation, 19 annotated endogenous differential metabolites were retained for downstream analysis, most of which belonged to lipid-related or amino-acid-related classes (Figure 3B; Supplementary Table 2). Correlation analysis also suggested links between part of this signature and clinical variables such as smoking exposure or body mass index (Figure 3C). Initial pathway enrichment highlighted arginine and proline metabolism, with additional signals in beta-alanine and histidine metabolism. These findings suggested that the salivary changes reflected broader metabolic remodeling (Figure 3D).

**FIGURE 3:**
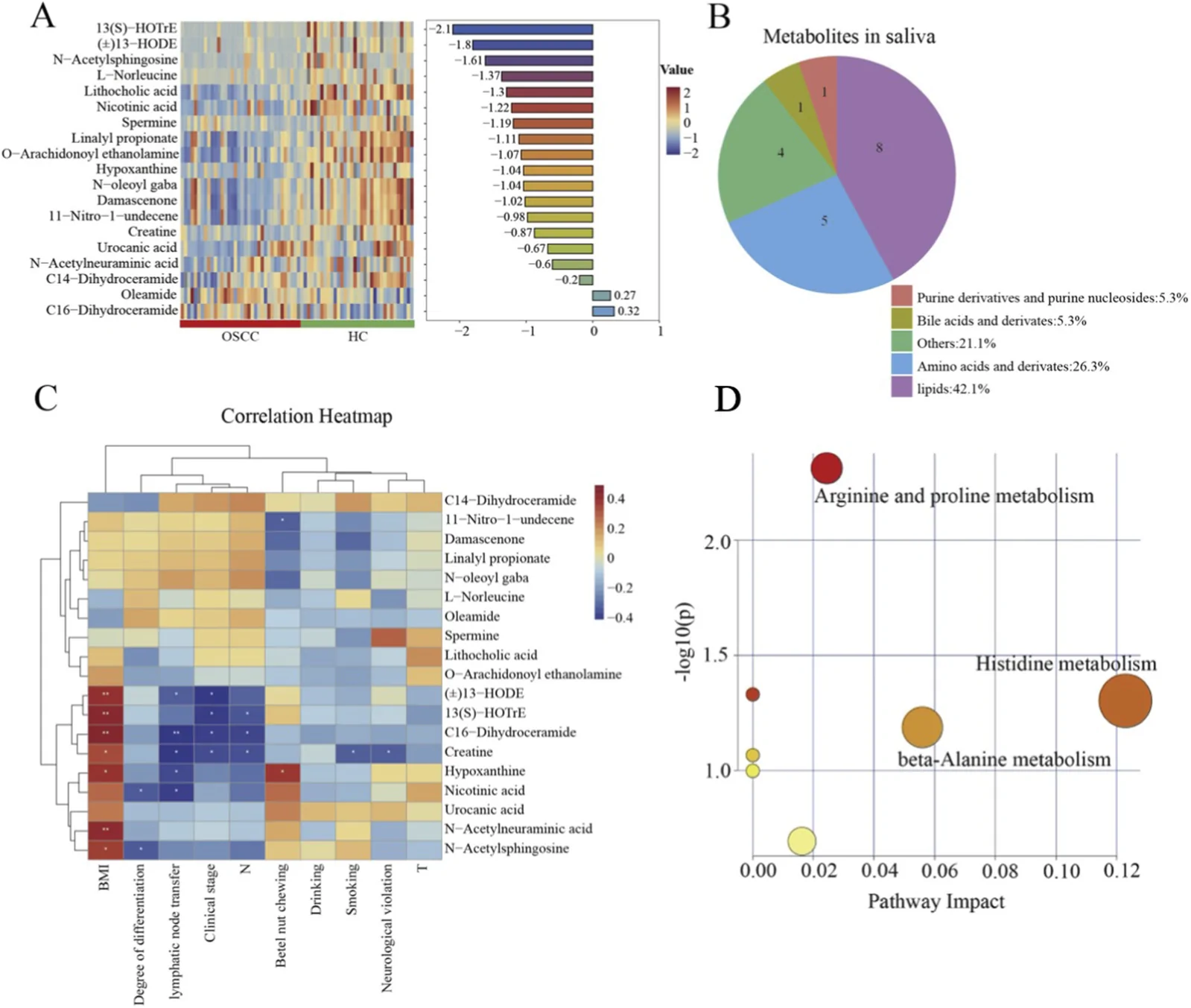
Differential salivary metabolites and their clinical associations. **(A)** Heatmap of the 19 endogenous differential metabolites identified in saliva samples from the OSCC and healthy control groups. **(B)** Fold-change summary of the differential metabolites. Negative values indicate lower abundance in OSCC, whereas positive values indicate higher abundance in OSCC. **(C)** Correlation heatmap showing the associations between differential metabolites and selected clinical variables. **(D)** Bubble plot of the enriched metabolic pathways derived from the salivary differential metabolite set. Bubble size represents pathway impact. The y-axis shows -log10(P value). Arginine and proline metabolism was the most prominent enriched pathway, with additional signals in beta-alanine metabolism and histidine metabolism.

### 3.3 A salivary metabolite-based classifier discriminated OSCC from controls

We next tested whether the differential metabolites could support a compact diagnostic model within the discovery cohort. We combined SVM-RFE ranking, random forest importance, and individual ROC performance across the 19-metabolite set (Figures 4A–C). This strategy repeatedly converged on seven metabolites: Damascenone, Linalyl propionate, O-arachidonoyl ethanolamine, 11-Nitro-1-undecene, N-oleoyl gaba, Lithocholic acid, and (±)13-HODE (Figure 4D). SVM-RFE ranking, random forest importance, and individual ROC performance across the 19-metabolite set were compiled in Supplementary Table S3, with the corresponding results shown in Figures 4E,F.

**FIGURE 4:**
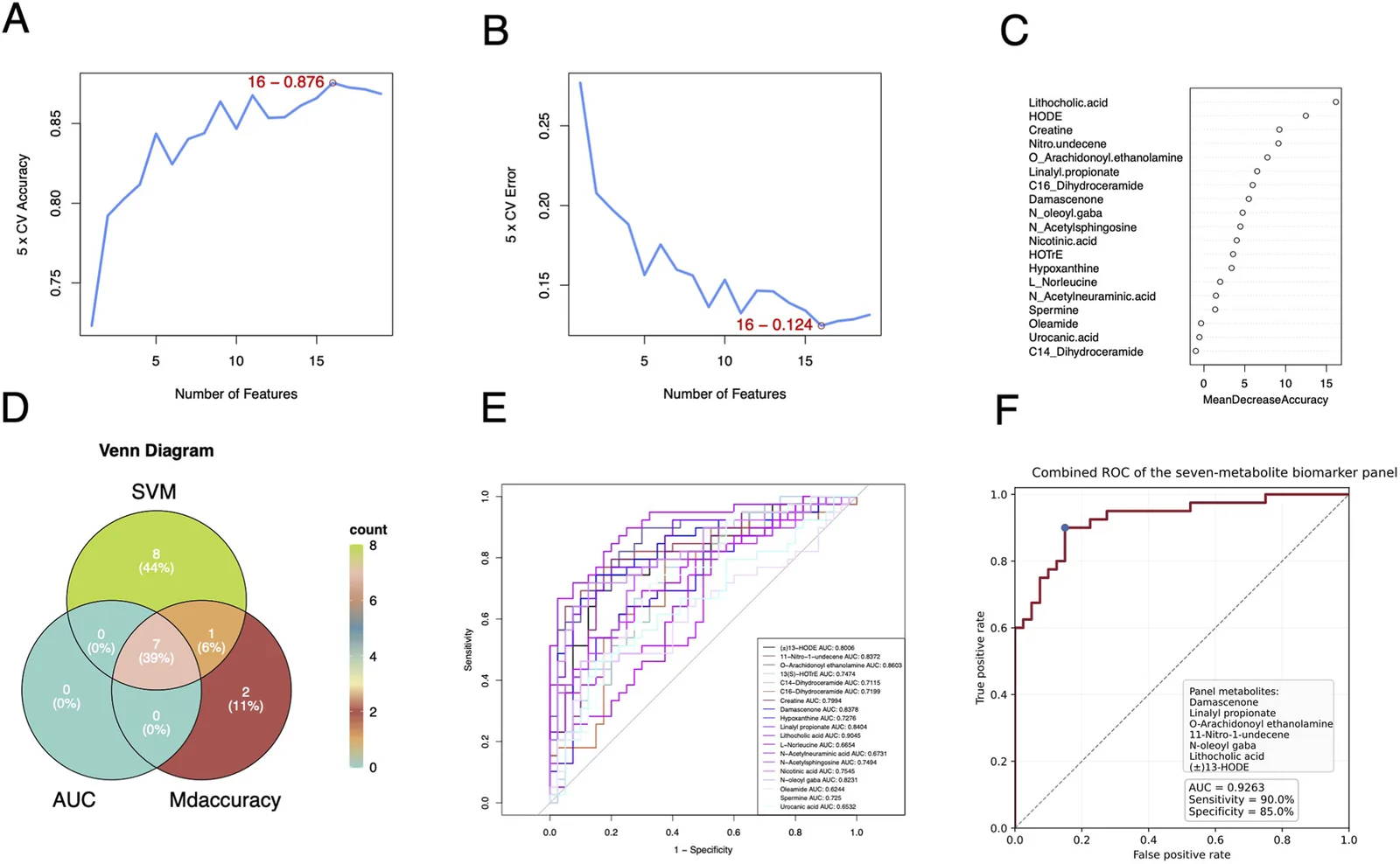
Screening candidate saliva metabolites through three algorithms. **(A)** SVM model accuracy plot across feature sets. **(B)** SVM model error plot across feature sets. **(C)** Random forest variable importance plot for the differential metabolite set. **(D)** Venn diagram showing the overlap among SVM-RFE ranking, random forest importance, and individual ROC performance. **(E)** ROC curves of the selected candidate metabolites. **(F)** ROC curves of seven candidate metabolites as biomarkers.

### 3.4 Candidate salivary biomarkers converged on coordinated metabolic programs

The seven candidate metabolites were chemically diverse, but they pointed to related biological processes. Lithocholic acid linked the signature to bile-acid-related signaling. O-arachidonoyl ethanolamine and N-oleoyl gaba pointed to lipid signaling and endocannabinoid-like metabolism. (±)13-HODE and 11-Nitro-1-undecene suggested lipid peroxidation and nitro-oxidative stress. Together with the broader metabolite set, these candidates converged on connected themes including bile-acid response, lipid remodeling, fatty acid metabolism, redox balance, and xenobiotic handling. Based on these metabolite classes and literature-guided biological themes, we explored five hypothesis-guided curated metabolic modules in downstream analyses: Lipid_Oxidation_Ferroptosis, Endocannabinoid_Signaling, Bile_Acid_Response, Nitro_Oxidative_Stress, and Xenobiotic_Metabolism. These modules were designed *post hoc* to summarize the observed metabolite patterns and should be interpreted as exploratory rather than as pre-registered hypotheses.

### 3.5 External transcriptomic datasets provided pathway-level concordance

To determine whether the salivary signature reflected underlying tissue-level metabolic alterations, we evaluated relevant pathway scores in two independent public transcriptomic datasets: GSE6631 and GSE37991. In both cohorts, several lipid- and bile-acid-related pathways tended to show lower scores in tumor tissue than in non-tumor tissue. The most recurrent patterns involved linoleic acid metabolism, arachidonic acid metabolism, primary bile acid biosynthesis, bile acid and bile salt metabolism, fatty acid metabolism, and the peroxisome pathway (Figure 5A). Additionally, glutathione metabolism was significantly reduced in tumors within GSE37991; a similar trend was observed in GSE6631, although the difference did not reach comparable significance. Nominal P values and FDR-adjusted q values for all pathway comparisons are provided in Supplementary Table 4; pathways that remained significant after FDR correction (q < 0.05) are distinguished from those showing only nominal trends (q ≥ 0.05).

**FIGURE 5:**
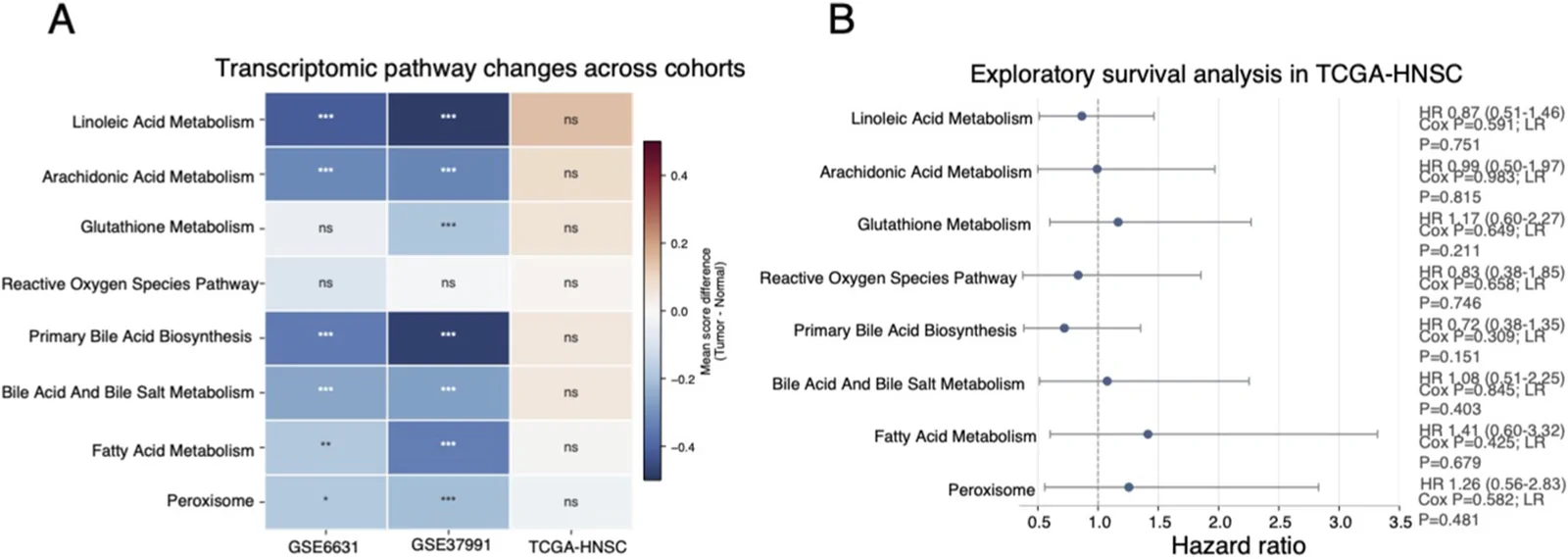
Salivary metabolic characteristics and exploratory survival analysis of TCGA-HNSC. **(A)** Heatmap summarizing pathway score differences between tumor and non-tumor tissues across GSE6631, GSE37991, and TCGA-HNSC. Values represent tumor minus normal mean scores. Pathway scores were compared between tumor and control groups within each cohort (GSE6631, GSE37991, and TCGA-HNSC) using the Mann–Whitney U test to obtain raw p values. Benjamini–Hochberg false discovery rate correction was then applied within each cohort across all pathway comparisons to obtain p_adj . Significance annotations (*, **, ***) were based on p_adj thresholds of <0.05,<0.01,<0.001 , respectively. **(B)** Forest-style summary of exploratory overall survival associations for representative pathways in TCGA-HNSC. hazard ratios and 95% confidence intervals were estimated using Cox proportional hazards models (Wald test), and survival differences between high- and low-expression groups were assessed using the log-rank test. BH-FDR correction was applied separately across pathways for Cox-model p values and log-rank p values, yielding cox_p_adj and logrank_p_adj, and adjusted results were prioritized for reporting.

Taken together, these transcriptomic findings provided pathway-level concordance with the salivary metabolite signal rather than direct validation of the salivary biomarkers themselves. They supported the view that the saliva-derived metabolic pattern was directionally consistent with broader tissue-level metabolic remodeling in OSCC.

### 3.6 TCGA-HNSC provided limited support and exploratory survival context

To further assess the generalizability of these findings, we examined the same metabolic pathways in the TCGA-HNSC cohort. Compared with the two GEO datasets, effect sizes were generally smaller, and several associations were attenuated or partially reversed in direction (Figure 5A). In this setting, the TCGA-HNSC results were interpreted cautiously because the cohort includes multiple head and neck subsites beyond the oral cavity. An oral-cavity-restricted sensitivity analysis was not performed due to the complexity of subsite annotation in TCGA metadata; we note that future work could address this by filtering to oral-cavity cases using the available TCGA clinical annotation.

We further evaluated the prognostic relevance of the eight pathway scores with respect to overall survival in TCGA-HNSC tumors. No pathway showed robust prognostic significance after accounting for multiple testing (Supplementary Table 4), and this part of the analysis is therefore presented as exploratory only (Figure 5B). Overall, TCGA-HNSC is better interpreted here as a heterogeneous pan-HNSC reference than as an OSCC-specific validation cohort.

### 3.7 Single-cell transcriptomics suggested reduced activity of curated metabolic modules in tumor-associated states

To define the cellular context of the metabolic signature, we analyzed the GSE181919 single-cell transcriptomic dataset, the annotation of cell subpopulations is shown in Figure 6A. The tissue-state composition plot revealed marked differences in cell-type distribution among normal mucosa, leukoplakia, primary cancer, and lymph node metastasis samples (Figure 6B), The number of malignant cells in CA group is much higher than that in LN group (Figure 6D). In addition, changes in cell subpopulation abundance were also analyzed in populations infected with HPV (Figure 6C). We then examined the five curated metabolic modules across major cell populations and tissue states. In the full cell atlas, the Lipid_Oxidation_Ferroptosis, Bile_Acid_Response, Nitro_Oxidative_Stress, and Xenobiotic_Metabolism modules tended to exhibit lower activity in the malignant compartment relative to several non-malignant populations (Figure 6E).

**FIGURE 6:**
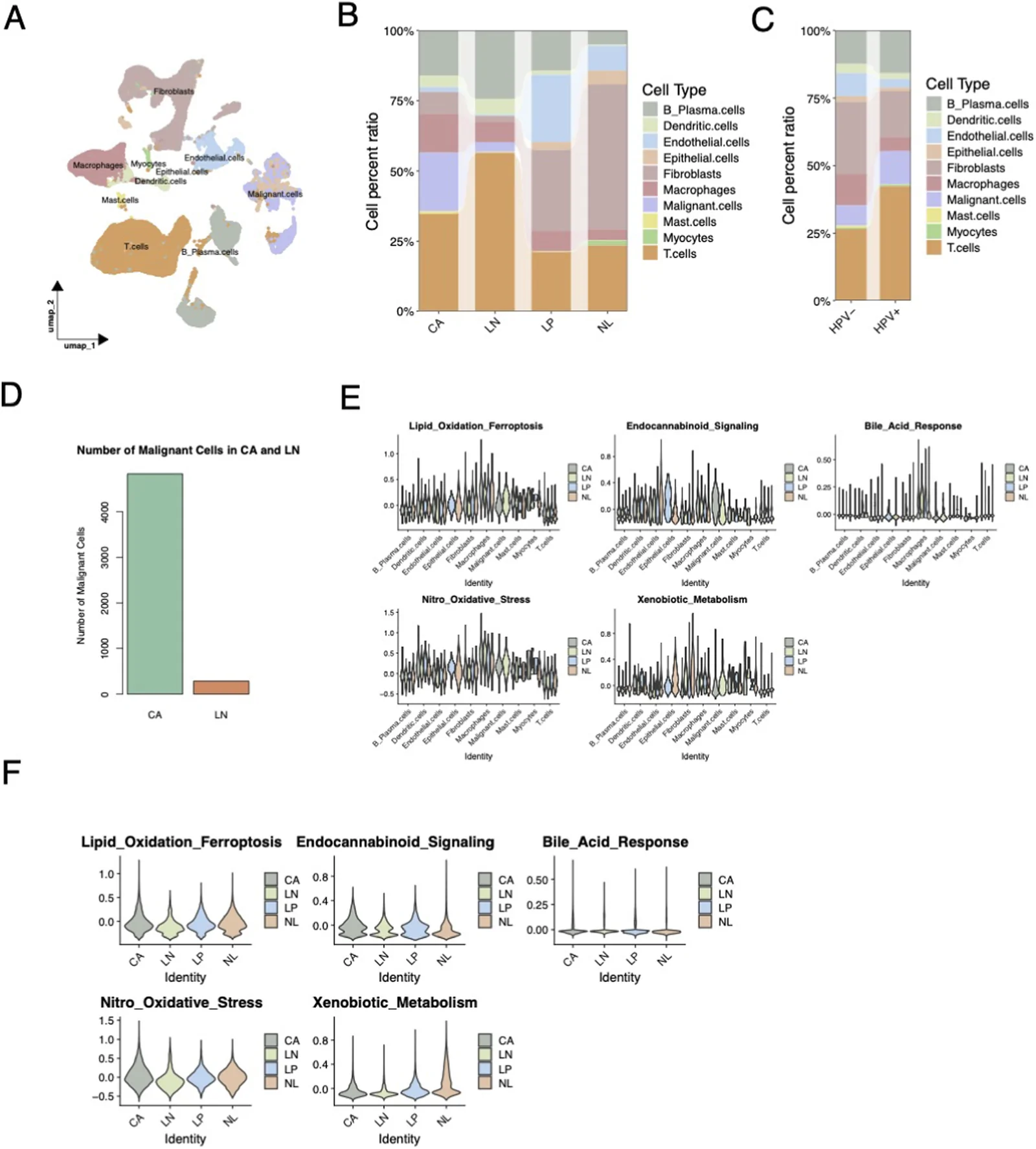
Single-cell transcriptomic analysis of curated metabolic modules. **(A)** UMAP visualization of the GSE181919 single-cell transcriptomic dataset showing the annotation and distribution of major cell populations. **(B)** Tissue-state composition plot showing the distribution of major cell populations across normal mucosa, leukoplakia, primary cancer, and lymph node metastasis samples. **(C)** Cell-type composition plot showing the distribution of major cell populations according to HPV status (HPV− and HPV+). **(D)** Bar plot showing the number of malignant cells in primary cancer (CA) and lymph node metastasis (LN) samples. **(E)** Module score plots across the full single-cell atlas for the five curated metabolic modules: Lipid_Oxidation_Ferroptosis, Endocannabinoid_Signaling, Bile_Acid_Response, Nitro_Oxidative_Stress, and Xenobiotic_Metabolism. **(F)** Module score plots restricted to malignant cells, showing the relative activity of the same modules across tissue states.

A similar pattern was seen when the analysis was restricted to malignant cells. Module activity was generally lower in primary cancer and lymph node metastasis samples than in leukoplakia or normal tissue, with the most notable reductions observed in xenobiotic metabolism, bile acid response, and lipid oxidation/ferroptosis-related programs (Figure 6F). These findings indicate that the salivary metabolic signature reflects tumor-associated metabolic reprogramming operating at the cellular level. We note that FDR correction was not applied to the single-cell analyses because the comparisons did not account for patient-level dependence, and we therefore describe these results qualitatively rather than relying on formal significance thresholds.

### 3.8 Protein-level analyses provided orthogonal support for representative pathway-related genes

To provide orthogonal validation at the protein level, we examined ALDH1A1 expression in the Human Protein Atlas (HPA) and quantified ALDH1A1, VDR, and SLC7A11 protein abundance in CPTAC proteomic data. In HPA immunohistochemistry, ALDH1A1 staining was predominantly localized to normal glandular and secretory structures, whereas tumor nests exhibited markedly weaker staining overall (Figure 7A). This pattern is compatible with reduced glandular or secretory differentiation in tumor tissue rather than necessarily reflecting tumor-intrinsic pathway suppression.

**FIGURE 7:**
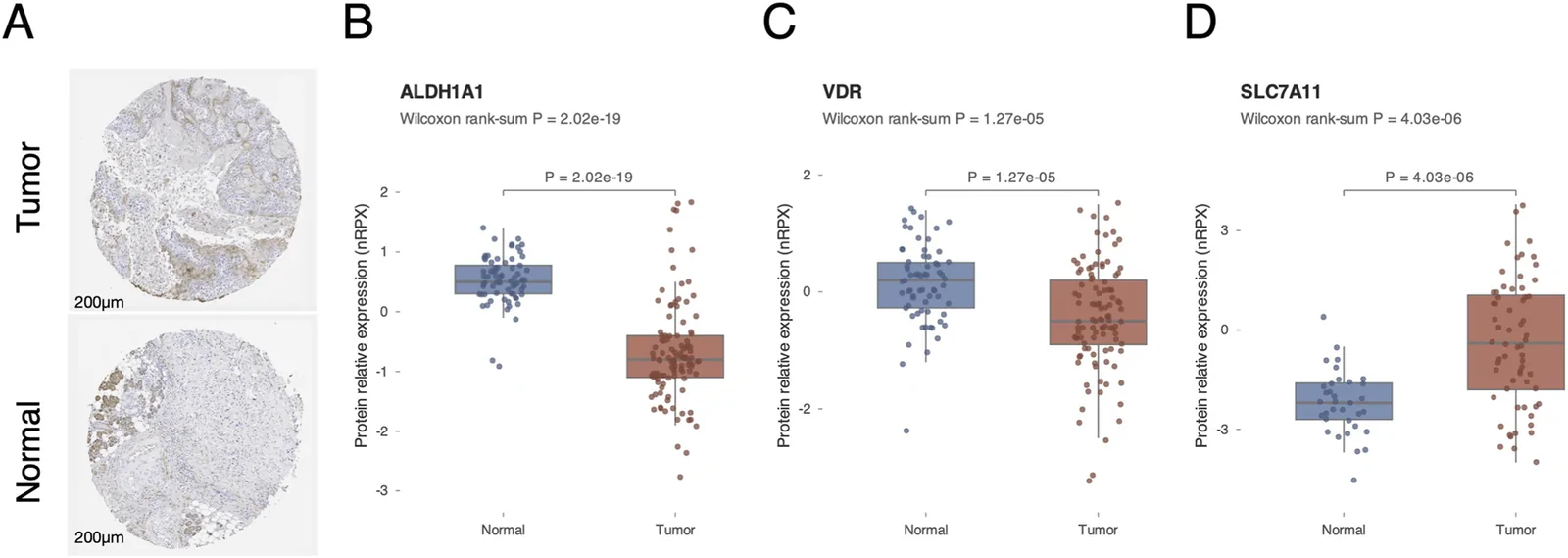
Protein-level difference of representative pathway-related genes. **(A)** HPA immunohistochemistry images of ALDH1A1 in normal and OSCC tissues, showing stronger staining in glandular or secretory structures of normal tissue and weaker overall staining in tumor nests. **(B)** Boxplot of ALDH1A1 protein abundance in tumor and normal tissues. **(C)** Boxplot of VDR protein abundance in tumor and normal tissues. **(D)** Boxplot of SLC7A11 protein abundance in tumor and normal tissues.

The CPTAC proteomic data showed lower ALDH1A1 and VDR abundance and higher SLC7A11 abundance in tumor tissue than in adjacent normal tissue (all nominal P < 0.001; Figures 7B–D; Supplementary Table 8). Given the small number of proteins tested (three), formal FDR correction was not applied. VDR showed a comparable reduction in tumors (tumor mean = −0.43; normal mean = 0.14; P = 1.27 × 10^−5^). In contrast, SLC7A11 protein levels were significantly elevated in tumor tissue (tumor mean = −0.37; normal mean = −2.19; P = 4.03 × 10^−6^) (Figure 7D). Collectively, these findings were directionally consistent with altered bile-acid-, xenobiotic-, and redox-related programs in tumor tissue.

### 3.9 Integrated analysis identified convergent metabolic signatures with biomarker potential in OSCC

Integration across data layers revealed several recurrent metabolic themes. First, bile-acid-related responses were directionally reduced across salivary metabolites, GEO transcriptomic pathway scores, single-cell module activity, and protein-level context for VDR. Second, lipid-associated metabolic pathways showed broadly concordant decreases across saliva, bulk transcriptomics, and single-cell analyses. Third, tumor tissue showed evidence consistent with enhanced redox defense, most notably reflected by higher SLC7A11 abundance (Figure 8).

**FIGURE 8:**
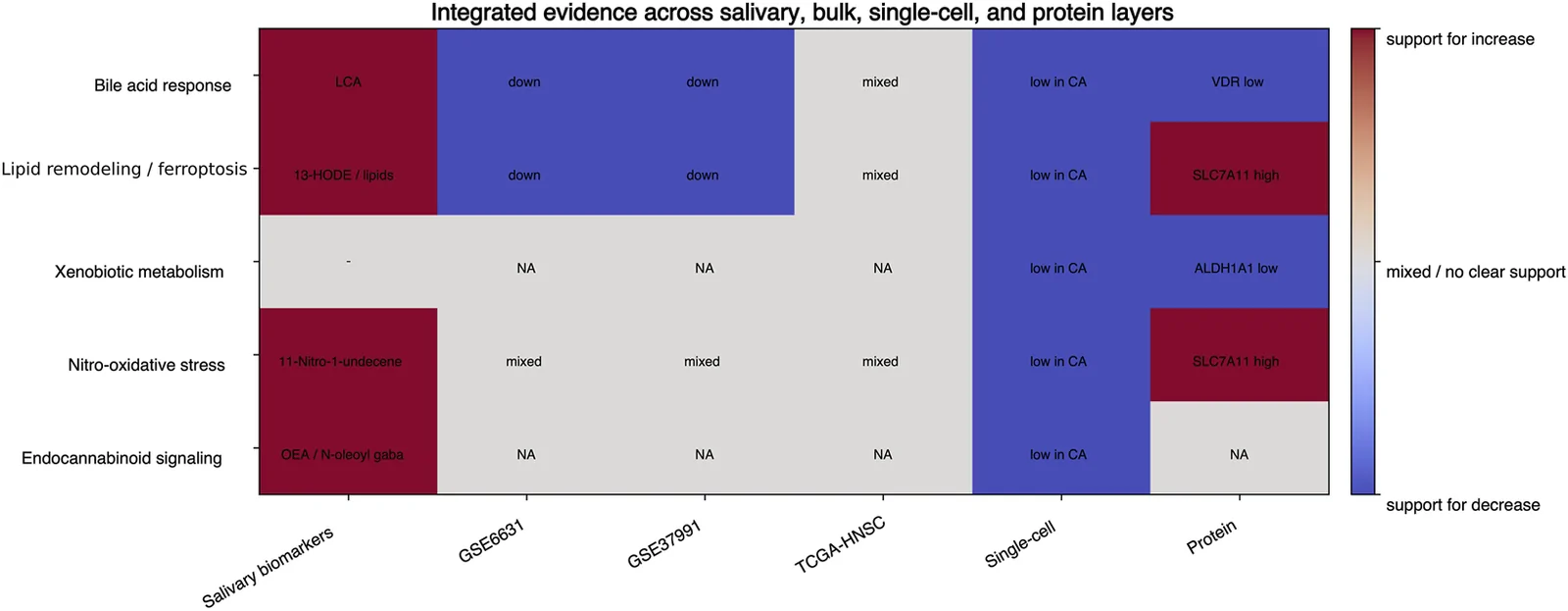
Integrated evidence summary across saliva, bulk transcriptomics, single-cell transcriptomics, and protein data**.** Summary heatmap integrating the main findings across all analytical layers. Rows represent major metabolic themes, including bile acid response, lipid remodeling or ferroptosis, xenobiotic metabolism, nitro-oxidative stress, and endocannabinoid signaling. Columns summarize support from salivary biomarkers, the two GEO cohorts, TCGA-HNSC, single-cell analysis, and protein validation.

## 4 Discussion

In this study, we combined salivary metabolomics with bulk transcriptomic, single-cell, and protein-level analyses to define a biologically coherent OSCC signature. The main findings can be summarized in three points. First, within the discovery cohort, salivary metabolomics supported a compact seven-metabolite candidate panel, although feature selection and evaluation were performed on the same samples without nested cross-validation. Second, these metabolites converged on a limited set of metabolic programs rather than representing unrelated changes. Third, related pathway-level patterns were directionally consistent across tissue, cellular, and protein-level data. Taken together, these results suggest that saliva may capture biologically relevant metabolic features of OSCC, while still requiring further targeted and external validation (Lau et al., 2024; Nijakowski et al., 2022).

These results resonate with a growing body of work on salivary biomarkers in oral cancer. Saliva has clear practical merits and non-invasive collection, low patient burden, easy repeat sampling, and these have been well documented in recent reviews (Lau et al., 2024; Nijakowski et al., 2022). Earlier salivary metabolomic studies showed that OSCC could be distinguished from controls by multi-metabolite patterns rather than by single compounds alone (Nijakowski et al., 2022; Ishikawa et al., 2016). In the present work, the combined panel outperformed the individual metabolites within the discovery cohort. This pattern is consistent with other metabolomic studies in OSCC and with broader experience in biomarker development, where panels usually outperform single markers because they better capture disease heterogeneity (Ishikawa et al., 2016; Gupta et al., 2024; Li et al., 2022; Hsu et al., 2019; Yang et al., 2020). However, because feature selection and evaluation were performed on the same 80 samples without nested cross-validation, the performance estimates are likely optimistic. In high-dimensional metabolomics studies with small sample sizes, internal feature selection without nested resampling can inflate AUC by 0.05–0.15 or more relative to out-of-sample performance; published benchmarks from comparable discovery-phase metabolomics studies suggest that optimism-corrected estimates should be the standard for reporting. In this study, we therefore refrain from reporting point estimates of AUC, sensitivity, or specificity, and instead present the panel as a candidate signature for future targeted validation.

One notable feature of our data was the prominence of lipid related signals. Several of the candidate metabolites belonged to lipid-associated classes or were linked to lipid signaling. The transcriptomic analyses then pointed in the same direction by showing lower activity of linoleic acid metabolism, arachidonic acid metabolism, fatty acid metabolism, and the peroxisome pathway in the two GEO cohorts. (±)13-HODE, a linoleic acid oxidation product, is of particular interest because its elevation can reflect both enzymatic lipoxygenase activity and non-enzyme. This interpretation is in line with recent reviews that describe metabolic rewiring in OSCC as a broad remodeling of lipid handling, oxidative balance, and bioenergetic flexibility (Gupta et al., 2024). It is also compatible with studies showing that lipid uptake-related pathways, including CD36-associated signaling, are linked to aggressive behavior in OSCC (Haidari et al., 2021). Although these observations do not establish a specific mechanism, they are consistent with the idea that altered lipid metabolism is an important component of the OSCC phenotype.

The bile acid related findings are also of interest. Lithocholic acid was one of the seven diagnostic metabolites, and both GEO cohorts showed lower scores for bile acid-related pathways in tumor tissue. In addition, VDR protein abundance was reduced in the CPTAC data. These layers point to a consistent weakening of bile acid-related signaling in OSCC. Lithocholic acid is a secondary bile acid of bacterial origin, which raises the possibility that its salivary reduction in OSCC partly reflects altered oral or gut microbial metabolism rather than—or in addition to—host metabolic changes. However, previous work has shown antineoplastic effects of lithocholic acid in other epithelial cancers, including pancreatic adenocarcinoma (Schwarcz et al., 2024). The oral microbiome was not profiled in this study, and this represents an important direction for future work seeking to disentangle host-derived from microbially derived metabolic signals. The present study does not establish a direct mechanistic role for lithocholic acid in oral cancer, and this interpretation should remain cautious.

The amino acid dimension of our data warrants particular attention. Metabolite enrichment analysis flagged arginine and proline metabolism as a leading pathway, and the broader differential profile included several additional amino acid-related compounds. This is not entirely surprising. Amino acid pathways in tumors do more than fuel biomass, they participate in redox buffering, mTOR signaling, and immune modulation (Lieu et al., 2020), roles that are increasingly recognized in head and neck cancer biology. Leung et al. reported arginine metabolism dysregulation in oral tongue squamous cell carcinoma (Leung et al., 2021), and Yang et al. demonstrated that amino acid-based panels could distinguish OSCC patients from controls (Yang et al., 2020). What our data add to this picture is context. The amino acid signal did not stand alone. Although the seven-metabolite candidate panel was dominated by lipid-related compounds, the broader 19-metabolite differential set included several amino acid derivatives, suggesting that amino acid and lipid alterations co-occur in the OSCC salivary metabolome. Further targeted quantification of amino acid pathways, alongside the lipid-focused candidates prioritized here, may strengthen the biological coverage of future salivary biomarker panels.

One feature of this study worth highlighting is that the analysis did not end with salivary metabolites. We pushed the interpretation outward—first into public transcriptomic datasets, then into single-cell data and protein-level evidence—to test whether the salivary signal had tissue-level backing. The two GEO cohorts, both derived from oral cavity tumors, returned a coherent picture: pathway scores for bile acid, lipid, and xenobiotic programs were consistently lower in tumor tissue. TCGA-HNSC did not replicate this pattern with the same clarity. Effect sizes were smaller, and some associations moved in the opposite direction. Why the discrepancy? The most parsimonious explanation is anatomical heterogeneity. The GEO datasets used here sampled oral cavity tumors specifically, whereas TCGA-HNSC aggregates head and neck cancers from the oral cavity, oropharynx, larynx, and hypopharynx subsites that differ in etiology, HPV status, and metabolic profile. When tumor biology is this mixed, pathway-level signals derived from a narrowly defined disease subset can easily be diluted. Others working on multi-cohort integrative analyses of OSCC have encountered comparable attenuation in pan-HNSC datasets (Gupta et al., 2024; Li et al., 2022; Hsu et al., 2019; Yang et al., 2020; Lieu et al., 2020; Leung et al., 2021). We therefore treat TCGA-HNSC not as a failed replication but as a useful counterpoint: it shows what happens when the signal is tested outside its biological range, and it reinforces rather than undermines the oral cavity specificity of our findings.

The single-cell and protein-level analyses added additional context but should also be interpreted cautiously. The single-cell module analyses were exploratory, performed at the individual cell level without accounting for patient-level dependence. Because hundreds to thousands of cells originate from a small number of patients, cell-level *P* values can be severely inflated. Module activities were lower in malignant cells and tumor-associated tissue states, suggesting that the salivary signal captures tumor-intrinsic metabolic reprogramming rather than a bulk tissue compositional artifact. At the protein level, ALDH1A1 and VDR were reduced in tumors, while SLC7A11 was elevated a pattern that makes biological sense. SLC7A11 controls cystine import and glutathione synthesis, and its upregulation in OSCC has been linked to ferroptosis resistance and tumor progression (Sun et al., 2024). The ALDH1A1 finding is less straightforward. ALDH1A1 is commonly treated as a stemness marker in head and neck cancer, yet its expression is context-dependent and serves multiple roles beyond stemness (Leinung et al., 2015). The lower overall protein abundance we observed in tumor tissue likely reflects loss of normal epithelial or secretory differentiation rather than a direct challenge to the stem-cell literature.

A further consideration in interpreting the candidate metabolite panel is the mixed provenance of the individual compounds. Damascenone and Linalyl propionate are primarily known as plant-derived flavor and fragrance constituents, and their presence in saliva may partially reflect dietary intake, oral hygiene product use, or environmental exposure rather than endogenous metabolic dysregulation. This does not necessarily negate their candidacy as biomarkers—exogenous compounds that differ systematically between cases and controls can still carry discriminatory information—but it complicates the biological narrative. Future studies aiming to link the salivary panel to tumor metabolism should include targeted assays that distinguish endogenous from exogenous metabolites, dietary and lifestyle questionnaires to assess potential sources of exogenous compounds, and, where feasible, stable-isotope tracer studies to confirm metabolic origin.

Several limitations should be explicitly acknowledged. First, feature selection and model evaluation for the seven-metabolite panel were performed on the same discovery cohort (n = 80) without nested cross-validation; therefore, the discriminatory performance estimates are likely optimistic and should not be interpreted as validated diagnostic accuracy. Second, formal multivariable adjustment for potential confounders—including smoking, BMI, alcohol use, betel-nut exposure, age, and sex—was not performed due to limited individual-level covariate data in this discovery cohort. Third, the salivary cohort was small and derived from a single center, and no independent salivary validation cohort was available. Fourth, the transcriptomic and proteomic datasets were not matched to the salivary samples, precluding direct patient-level inference. Fifth, TCGA-HNSC is biologically heterogeneous, and an oral-cavity-restricted sensitivity analysis was not performed. Sixth, the single-cell analyses did not account for patient-level dependence through pseudobulk or mixed-effects modeling. Seventh, the curated metabolic modules were defined *post hoc* based on the observed metabolite classes, which carries a risk of confirmatory bias; future studies should include parallel analyses using predefined pathway databases (e.g., KEGG, Reactome, MSigDB). Eighth, potential residual confounding by lifestyle and host factors warrants more extensive assessment in larger cohorts with comprehensive covariate data. Ninth, several candidate metabolites have known exogenous sources (dietary, environmental), and the relative contribution of endogenous versus exogenous origins was not experimentally determined. Tenth, the oral microbiome was not profiled; microbially derived metabolites (particularly lithocholic acid) may contribute to the salivary signature independently of host tumor metabolism (Gupta et al., 2024).

Within these constraints, the main contribution of the study is the convergence of several metabolic themes across independent analytical layers. Rather than claiming direct diagnostic validation, the present work helps prioritize candidate metabolites and pathways for future targeted quantification, paired saliva-tissue studies, controlled dietary assessment, microbiome profiling, and mechanistic follow-up.

## 5 Conclusion

In summary, we identified a saliva-derived metabolic signature associated with OSCC and supported by pathway-level concordance across transcriptomic, single-cell, and protein-level data. The most recurrent themes involved lipid remodeling, bile-acid-related signaling, amino acid metabolism, and redox defense. The seven-metabolite panel was derived and evaluated in the same discovery cohort without nested cross-validation or external validation; accordingly, performance metrics are not reported as validated diagnostic estimates, and the findings should be interpreted as hypothesis-generating. These observations support further targeted quantification, controlled assessment of exogenous metabolite sources, and external validation of saliva-based biomarker candidates for OSCC.

## Data Availability

The datasets presented in this study can be found in online repositories. The names of the repository/repositories and accession number(s) can be found in the article/Supplementary Material.
