# Supplementary material for: A saliva-derived metabolic signature with multi-omics support for oral squamous cell carcinoma diagnosis
Source: Front Cell Dev Biol. 2026 Sep 25;14:1901210. doi: 10.3389/fcell.2026.1901210 (PMC13649689; doi:10.3389/fcell.2026.1901210)
Supplement: Supplementary file 1 [file Table3.DOCX]

| No. | Retention time(min) | Metabolite name | Formula | Molecular weight | (M+H)^+^/ (M-H)^-^ | Fragments | Fragments | Fragments | Expression level |
| --- | --- | --- | --- | --- | --- | --- | --- | --- | --- |
| 1 | 0.61 | Spermine | C_10_ H_26_ N_4_ | 203.22316 | [M+H]+1 | 129.14 | 112.11 | 203.22 | Down |
| 2 | 0.85 | Creatine | C_4_ H_9_ N_3_ O_2_ | 132.07677 | [M+H]+1 | 90.10 | 132.10 | 115.10 | Down |
| 3 | 0.87 | N-Acetylneuraminic acid | C_11_ H_19_ N O_9_ | 310.11356 | [M+H]+1 | 274.50 | 214.00 | 98.80 | Down |
| 4 | 0.91 | Urocanic acid | C_6_ H_6_ N_2_ O_2_ | 139.05017 | [M+H]+1 | 139.05 | 121.04 | 93.05 | Down |
| 5 | 0.91 | Hypoxanthine | C_5_ H_4_ N_4_ O | 137.04579 | [M+H]+1 | 137.05 | 69.10 | 121.03 | Down |
| 6 | 0.96 | Nicotinic acid | C_6_ H_5_ N O_2_ | 124.03944 | [M+H]+1 | 124.04 | 80.05 | 96.04 | Down |
| 7 | 1.17 | L-Norleucine | C_6_ H_13_ N O_2_ | 132.1019 | [M+H]+1 | 132.10 | 86.10 | 132.10 | Down |
| 8 | 7.1 | N-OLEOYL-4-AMINOBUTYRIC ACID | C_22_ H_41_ N O_3_ | 368.31363 | [M+H]+1 | 368.31 | 350.30 | 228.20 | Down |
| 9 | 8.67 | 13-HODE | C_18_ H_32_ O_3_ | 295.22803 | [M-H]-1 | 59.01 | 113.10 | 277.22 | Down |
| 10 | 8.87 | 13(S)-HOTrE | C_18_ H_30_ O_3_ | 293.21259 | [M-H]-1 | 221.15 | 195.17 | 293.21 | Down |
| 11 | 9.09 | Lithocholic acid | C_24_ H_40_ O_3_ | 377.3035 | [M+H]+1 | 377.30 | 359.29 | 315.27 | Down |
| 12 | 9.11 | N-Acetylsphingosine | C_20_ H_39_ N O_3_ | 342.30048 | [M+H]+1 | 341.27 | 57.07 | 71.09 | Down |
| 13 | 9.74 | O-Arachidonoyl ethanolamine | C_22_ H_37_ N O_2_ | 348.28732 | [M+H]+1 | 348.28 | 199.17 | 90.98 | Down |
| 14 | 9.83 | C16-Dihydroceramide | C_34_ H_69_ N O_3_ | 540.53619 | [M+H]+1 | 540.29 | 284.29 | 523.42 | Up |
| 15 | 10.14 | 11-Nitro-1-undecene | C_11_ H_21_ N O_2_ | 200.16449 | [M+H]+1 | 107.08 | 95.08 | 55.05 | Down |
| 16 | 10.16 | C14-Dihydroceramide | C_32_ H_65_ N O_3_ | 512.50439 | [M+H]+1 | 512.50 | 283.26 | 212.23 | Down |
| 17 | 10.37 | Oleamide | C_18_ H_35_ N O | 282.27911 | [M+H]+1 | 109.10 | 97.10 | 83.09 | Up |
| 18 | 10.64 | Damascenone | C_13_ H_18_ O | 191.14317 | [M+H]+1 | 149.09 | 121.10 | 105.07 | Down |
| 19 | 10.75 | Linalyl propionate | C_13_ H_22_ O_2_ | 211.16937 | [M+H]+1 | 155.10 | 57.07 | 69.07 | Down |
